# *In vitro* inhibition of human red blood cell acetylcholinesterase (AChE) by temephos-oxidized products

**DOI:** 10.1038/s41598-019-51261-2

**Published:** 2019-10-14

**Authors:** Francisco Alberto Verdín-Betancourt, Mario Figueroa, Ma. de Lourdes López-González, Elizabeth Gómez, Yael Yvette Bernal-Hernández, Aurora Elizabeth Rojas-García, Adolfo Sierra-Santoyo

**Affiliations:** 1 Departamento de Toxicología, Centro de Investigación y de Estudios Avanzados del Instituto Politécnico Nacional (Cinvestav-IPN), Col. San Pedro Zacatenco, Ciudad de México, C.P. 07360 Mexico; 2 Facultad de Química, Universidad Nacional Autónoma de México, Ciudad de México, 04510 Mexico; 3 Instituto de Química, Universidad Nacional Autónoma de México, Ciudad de México, 04510 Mexico; 4 Laboratorio de Contaminación y Toxicología Ambiental, Universidad Autónoma de Nayarit, Tepic, Nayarit 63155 Mexico

**Keywords:** Chemical tools, Risk factors

## Abstract

Temephos (Tem) is an organophosphorus pesticide widely used to kill and prevent the growth of the main vectors for the transmission of dengue, zika, and chikungunya viruses. In chlorinated water, Tem is oxidized to its dioxon-sulfoxide (Tem-dox-SO), dioxon-sulfone (Tem-dox-SO_2_), and sulfoxide (Tem-SO) derivatives; however, these compounds are not commercially available to be used as standards and in toxicological studies. In the present study, we synthesized and characterized the Tem-oxidation products and the compound 4,4′-sulfinyldiphenol. These compounds were obtained by a simple reaction between Tem or 4,4′-thiodiphenol with sodium hypochlorite or potassium periodate, and were characterized by IR, NMR, and UPLC-HRESIMS. The *in vitro* evaluation of inhibitory potency of Tem-oxidized products on human red blood cell acetylcholinesterase (RBC AChE) showed that Tem-dox-SO_2_ was the most potent inhibitor of human RBC AChE, and its effect was more pronounced than that observed for ethyl-paraoxon, a potent typical inhibitor of AChE. An HPLC-DAD method for the analysis of metabolic products of Tem was developed, which may be useful for monitoring in biological and environmental samples. The ability of Tem-oxidized metabolites to inhibit human RBC AChE suggests that the addition of Tem to chlorinated drinking water could result in an increase in the risk of RBC AChE inhibition after exposure.

## Introduction

Temephos (*O*,*O*,*O*′,*O*′-tetramethyl *O*,*O*′-thiodi-*p*-phenylene bis(phosphorothionate); Tem) is an organophosphorus insecticide recommended by the World Health Organization (WHO) for the control of mosquitoes, midges, black flies, fleas, and other insects at concentrations not exceeding 1 mg/L^1^. The pesticide, developed by American Cyanamid between 1963 and 1967^2^ is particularly important for killing the larvae of *Aedes aegypti*, the transmitting vector of dengue, zika, and chikungunya viruses. In countries where these diseases represent a public health problem, Tem is utilized in a massive and permanent manner. For example, in Mexico, it is distributed in plastic bags for its application in household water containers and is also used in ponds, lakes, lagoons, and other bodies of water that serve as breeding grounds for mosquito larvae^3,4^.

**Figure 1:**
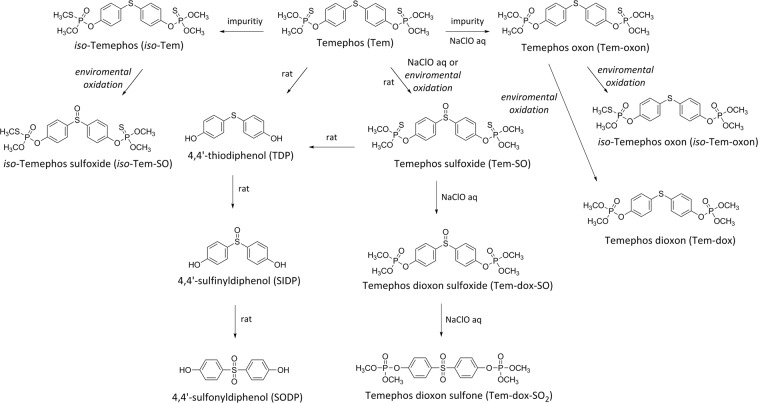
Chemical structures of Tem common impurities, and metabolism and degradation products.

The most relevant impurities of technical grade Tem are its oxon derivative (Tem-oxon) and an isomer (*iso*-Tem)^5^. Early investigations have shown that Tem is transformed by chemical oxidation, photolysis, or metabolism by mammals and insects (Fig. 1)^6–8^. Kamel *et al*. reported that, after 72 h of incubation in chlorinated water, Tem undergoes oxidation to form stable products: sulfoxide (Tem-SO), dioxon-sulfoxide (Tem-dox-SO), and dioxon-sulfone (Tem-dox-SO_2_) (Fig. 1)^9^. When orally administered in rats, Tem is rapidly absorbed (at least 40%) into the bloodstream; Tem-SO, 4,4′-thiodiphenol (TDP), 4,4′-sulfinyldiphenol (SIDP), and 4,4′-sulfonyldiphenol (SODP) or bisphenol S (BPS) were identified as its main metabolites in the urine (Fig. 1)^6^.

Temephos is considered innocuous for humans^1^, although its toxicological information in mammals is limited, and for its oxidized products are unknown. In addition, there is currently no data on the dosimetry of Tem and its metabolites for establishing their dose-response and toxicological parameters, and no acceptable daily intake or reference dose has been established because the majority of these studies were of poor quality because they did not comply with good laboratory practices (GLP)^1,10^. This type of studies are necessary for the appropriate risk assessment of exposed humans to Tem via drinking water.

Moreover, information on its complete biotransformation, distribution, accumulation, and the toxicity of its metabolites is very limited. The biotransformation of phase I of pesticides is relevant because some organophosphates need to be bioactivated to produce the desired effects on insects, and sometimes, the metabolic products are more toxic than the parent compound^11^. At present, there is, to our knowledge, no information to clarify any relationship between metabolites of Tem and human red blood cell acetylcholinesterase (RBC AChE) inhibition. It is important to evaluate the inhibition of cholinesterases (AChE and butyrylcholinesterase (BuChE)) activities by the products generated by the oxidation of Tem in biotic and abiotic systems, as an indicator of possible toxicological hazard.

Despite the widespread use of Tem, the analysis of Tem and its metabolites in environmental, food, water, and biological samples has received limited attention, in part is due to the limited availability of standards. Therefore, the aim of the present study was to synthesize and obtain four potentially toxic Tem-oxidized metabolites, to evaluate their effect on human RBC AChE activity and then to develop an HPLC-DAD analytical method for the analysis of Tem and its metabolites.

## Materials and Methods

### Reagents

Temephos, TDP, SODP, potassium periodate, acetylthiocholine iodide (ATCh), 5,5-dithio-2-*bis*-nitrobenzoic acid (DTNB), Triton X-100, butyrylthiocholine, and ethopropazine hydrochloride were purchased from Sigma-Aldrich (St. Louis, MO, USA). Ethyl-paraoxon was obtained from Supelco. T.M. FOS^®^ 500 CE (46%) was acquired from Agromundo, S.A. de C.V. (Mexico City, Mexico). HPLC-grade methanol, ethyl acetate, hexane, and acetonitrile (ACN) were purchased from Fisher Chemical (Fair Lawn, NJ, USA). Ultrapure water was obtained from a Milli-Q system (Millipore, USA). MS-grade water and acetonitrile were purchased from J.T. Baker (Center Valley, PA, USA). Deuterated NMR solvents were acquired from Cambridge Isotope Laboratories (Tewksbury, MA, USA). Sodium hypochlorite (Cloralex^®^, 5% chlorine solution) was acquired from Alen del Norte, S.A. de C.V. (Monterrey, NL, Mexico). The Drabkin reagent was obtained from Hycel (Houston, TX, USA). All other chemicals were of the highest purity available.

### Purification of temephos

Temephos was purified from the commercial product T.M. FOS^®^ 500 CE. For this, 0.5 L of T.M. FOS^®^ 500 CE was placed in a beaker and the volatile excipient was evaporated under a stream of air. The residue was dissolved in acetonitrile, stirred, allowed to precipitate for 5 min, and then centrifuged for 10 min at 3,000 rpm. The supernatant was stored at 4 °C. This process was repeated until no precipitate was observed. Then, the supernatant (Tem in ACN) was washed with hexane to remove any other components from the excipient. The solvent was evaporated and the residue was analyzed by HPLC-DAD. The purified Tem was stored in the dark at room temperature until its use.

### NMR, IR and HRESIMS analysis

IR spectra were obtained using a Bruker Tensor 27 spectrometer (Billerica, MA, USA). UV-Visible spectra were acquired with a Genesys 10S spectrophotometer (Thermo Fisher Scientific, Waltham, MA, USA). NMR data were collected with a Bruker Advance III spectrometer at 300.0 (^1^H), 75.4 (^13^C), and 121.4 (^31^P) MHz in CD_3_OD or CDCl_3_. UPLC-HRESIMS data were measured using a Q-Exactive system (Thermo Fisher Scientific) equipped with an electrospray ionization source, in both positive and negative ion modes, via an Acquity UPLC system (Waters, Milford, MA, USA). For the UPLC-MS analysis, a BEH C18 column (Waters) (50 × 2.1 mm i.d., 1.7 μm) was used with a gradient solvent system from 20:80 to 100:0 ACN:H_2_O (0.1% formic acid) in 10 min.

### Synthesis, purification, and identification of temephos-oxidized products

Temephos-oxidized derivatives were prepared as follows. (A) Tem and NaClO (5%), at a 1:10 or 1:100 molar ratio, were stirred during 2 min at room temperature. The reaction products were extracted with ethyl acetate, which was dried using anhydrous sodium sulfate and then evaporated with a stream of N_2_. The extract was dissolved in methanol and purified by flash chromatography. Flash chromatography was carried out on a CombiFlash Rf+ Lumen system equipped with a photo diode array (PDA) and evaporative light-scattering (ELS) detectors, and using a RediSep Rf High-Performance C18 column (150 g, particle size 20–40 μm, 100 Å) (Teledyne Technologies, Inc., Lincoln, NE, USA). The mobile phase consisted of water (A) and ACN (B). The gradient conditions changed from 80% A and 20% B to 43.5% A and 56.5% B in 6.9 min, and then to 100% of B in 6 min at a flow rate of 85 mL/min. Fractions were collected every 23 mL and pooled into eight fractions according to their UV and ELSD profiles. The purity of Tem-dox-SO and Tem-dox-SO_2_ was verified by HPLC-DAD analysis.

(B) Equivalent amounts (0.5 g) of Tem or TDP and potassium periodate were stirred in 100 mL of methanol during 24 h at room temperature. Then, the residue was dissolved in 20 mL of water, the reaction products were extracted twice with 100 mL ethyl acetate, and the solvent was dried with anhydrous sodium sulfate and evaporated with a stream of N_2_. The extract was dissolved in methanol and purified simply by washing with organic solvents: Tem-SO was extracted with hexane:toluene (2:1, v/v), and SIDP, with hexane:ethyl acetate (2:1, v/v). The purity of the compounds was verified by HPLC-DAD analysis and the chemical identity of compounds was performed by IR, ^1^H, ^13^C, and ^31^P NMR, and HRESIMS.

#### Tem-dox-SO

yellow oil; IR ν_max_: 1586 (C=C), 1278 (P=O), 1165 (P-O-CH_3_), 1035 (S=O) cm^−1^; ^1^H NMR (CDCl_3_): *δ*_H_ 3.85 (6H, d, ^3^*J* (^1^H-^31^P) = 11.4 Hz, CH_3_), 7.35 (2H, d, *J* = 8.4 Hz, H-3, H-5), 7.92 (2H, d, *J* = 8.7 Hz, H-2, H-6); ^13^C NMR (CDCl_3_) *δ*_C_ 55.2 (d, ^2^*J* (^13^C-^31^P) = 6.0 Hz, CH_3_), 121.6 (d, ^3^*J* (^13^C-^31^P) = 5.3 Hz, C-3, C-5), 129.9 (C-2, C-6), 138.0 (C-1), 154.3 (d, ^2^*J* (^13^C-^31^P) = 6.0 Hz, C-4); ^31^P NMR (CDCl_3_) *δ*_P_ −4.9; MS: HRESIMS *m/z* 451.0371 [M + H]^+^ (calculated for C_16_H_21_O_9_SP_2_ 451.0376, Δ = −1.1 ppm, IHD = 8).

#### Tem-dox-SO_2_

yellow oil; IR ν_max_: 1588 (C=C), 1283 (P = O), 1185 (P-O-CH_3_), 1153 (O=S=O) cm^−1^; ^1^H NMR (CDCl_3_): *δ*_H_ 3.85 (6H, d, ^3^*J* (^1^H-^31^P) = 11.4 Hz, CH_3_), 7.31 (2H, d, *J* = 8.4 Hz, H-3, H-5), 7.61 (2H, d, *J* = 8.7 Hz, H-2, H-6); ^13^C NMR (CDCl_3_): *δ*_C_ 55.1 (d, ^2^*J* (^13^C-^31^P) = 6.0 Hz, CH_3_), 121.0 (d, ^3^*J* (^13^C-^31^P) = 5.3 Hz, C-3, C-5), 126.6 (C-2, C-6), 141.7 (C-1); 152.7 (d, ^2^*J* (^13^C-^31^P) = 6.0 Hz, C-4); ^31^P NMR (CDCl_3_): *δ*_P_ −4.6; MS: HRESIMS *m/z* 467.0320 [M + H]^+^ (calculated for C_16_H_21_O_10_SP_2_ 467.0325, Δ = −1.1 ppm, IHD = 8).

#### Tem-SO

brown oil; IR ν_max_: 1585 (C=C), 1160 (P-O-CH_3_), 1025 (S=O), 648 (P=S) cm^−1^; ^1^H NMR (MeOH-*d*_4_): *δ*_H_ 3.83 (6H, d, ^3^*J* (^1^H-^31^P) = 13.8 Hz, CH_3_), 7.35 (2H, d, *J* = 8.7, H-3, H-5), 7.71 (2H, d, *J* = 8.7 Hz, H-2, H-6); ^13^C NMR (MeOH-*d*_4_) *δ*_C_ 54.6 (d, ^2^*J* (^13^C-^31^P) = 5.8, CH_3_), 122.0 (d, ^3^*J* (^13^C-^31^P) = 5.0 Hz, C-3, C-5), 126.4 (d, ^4^*J* (^13^C-^31^P) = 1.0 Hz, C-2, C6), 141.1 (d, ^5^*J* (^13^C-^31^P) = 1.5 Hz, C-1), 153.1 (d, ^2^*J* (^13^C-^31^P) = 7.2 Hz, C-4); ^31^P NMR (MeOH-*d*_4_) *δ*_P_ 66.1; MS: HRESIMS *m/z* 482.9915 [M + H]^+^ (calculated for C_16_H_21_O_7_S_3_P_2_ 482.9919, Δ = −0.9 ppm, IHD = 8).

#### SIDP

dark brown oil; IR ν_max_: 3152 (OH),1582 (C=C), 1010 (S=O), 1220 (C–OAr) cm^−1^; ^1^H NMR (MeOH-*d*_4_): *δ*_H_ 6.91 (2H, d, *J* = 8.7 Hz, H-3, H-5), 7.45 (2H, d, *J* = 8.7 Hz, H-2, H-6); ^13^C NMR (MeOH-*d*_4_): *δ*_C_ 116.0 (C-3, C-5), 127.15 (C-2, C-6), 133.7 (C-1), 160.6 (C-4); MS: HRESIMS *m/z* 235.0418 [M + H]^+^ (calculated for C_12_H_11_O_3_S 235.0423, Δ = −2.3 ppm, IHD = 8).

### Analysis of temephos and its metabolites by liquid chromatography

Temephos and its metabolites were analyzed by injecting samples into a liquid chromatograph equipped with a quaternary pump, autosampler, degaser, and a DAD (model 1200, Agilent Technologies, Palo Alto, CA, USA). ChemStation software was used for data acquisition and management of the chromatographic output. The detector wavelength was set at 254 nm and the reference wavelength was 550 nm. A ZORBAX Eclipse XDB-C18 column (4.6 × 150 mm, 5 μm) (Agilent Technologies, Deerfield, IL, USA) was used. The mobile phase consisted of water (A), methanol (B), and ACN (C) with initial solvent conditions of 60% A, 34% B, and 6% C at a rate of 1 mL/min at room temperature. After injecting the sample (10 μL), there was an 8 min linear gradient change to 20% A, 74% B, and 6% C followed by a second 3 min linear gradient change to 5% A, 65% B, and 30% C; then, the conditions were maintained for 2 min. The initial conditions were then re-established and the column was equilibrated for 5 min before the next injection. The identity of Tem and its metabolites was confirmed by their retention time (*t*_ret_) and UV spectra.

### Human blood sample

RBC AChE and BuChE assays were performed on whole human blood and serum, respectively. In this study, all methods were conducted in accordance with guidelines and regulations to comply with the GLP and chemical wastes management established by the Cinvestav-IPN. The sampling protocol was approved by the Bioethics Commission of Nayarit State, Mexico (CEBN/011/2017). The subject gave written informed consent for his participation and blood samples were collected early in the morning. A human blood sample was collected from a healthy adult male volunteer from Mexico City aged 28 years. The participant received medical examinations, and no issues with blood pressure, glucose and lipid blood levels, alcoholism, pathological antecedents, or medical treatments were detected.

### Acetylcholinesterase activity

RBC AChE activity was evaluated according to the method reported by Ellman *et al*.^12^ with some modifications^13^. Briefly, fresh human whole blood samples were diluted (1:100) with Triton X-100 (0.03% in phosphate buffer 0.1 M, pH 7.4). The enzymatic assay media consisted of 500 µL diluted blood, 1 mL of phosphate buffer 0.1 M, pH 7.4, 0.05 mL of DTNB (10 mM), and 5 µL of ethopropazine (6 mM), which were mixed and incubated at 37 °C for 10 min. The reaction was started by the addition of 25 µL of substrate ATCh (28.3 mM) and absorbance was monitored at 436 nm during 3 min with a UV-Vis spectrophotometer (Thermo Scientific GENESYS 10S). AChE activity was corrected for hemoglobin (Hb) content and was reported in U/g Hb. The Hb content was determined at 540 nm using the Drabkin reagent. The molar extinction coefficient of the TNB hydrolysis product was ε = 10,600 M^−1^ cm^−1^.

### Butyrylcholinesterase activity

Human BuChE activity was determined in serum according to the Ellman *et al*.^12^ method with slight modifications^13^. A mixture containing 0.1 mL of human serum, 3.0 mL of phosphate buffer (0.1 M, pH 7.4), and 0.10 mL of DTNB (10 mM) was incubated at 37 °C for 10 min. Then, 0.05 mL of butyrylthiocholine iodide (63.2 mM) was added. Changes in absorbance were monitored at 405 nm during 4 min with a UV-Vis spectrophotometer (Thermo Scientific GENESYS 10S). The activity is reported in U/L.

### Acetylcholinesterase and butyrylcholinesterase inhibition assays

To determine the inhibitory effect on human RBC AChE activity, the hemolyzed samples were preincubated with Tem and its metabolites for 15 min at 37 °C. The RBC AChE activity was evaluated as previously described by Bernal-Hernández *et al*.^13^ in three independent experiments conducted in duplicate. Stock solutions of Tem metabolites were individually prepared in ethanol (Tem-SO, 4.14 mM; Tem-dox-SO, 4.44 mM, and Tem-dox-SO_2_, 4.20 mM). These solutions were used to prepare other diluted solutions in phosphate buffer (0.1 M, pH 7.4) to achieve the following concentrations: 0.1 to 10 μM Tem-SO, 0.05 to 5 μM Tem-dox-SO, and 0.02 to 2 μM Tem-dox-SO_2_. Ethyl-paraoxon (0.01 to 1 μM) was used as the positive control of RBC AChE inhibition. To determine the inhibitory effect on human BuChE, the serum samples were previously incubated with Tem-dox-SO_2_ (0.02 to 2 μM) or ethyl-paraoxon (0.01 to 1 μM) during 10 min at 37 °C, and the enzymatic activity was evaluated as previously described, in three independent experiments conducted in duplicate. Log IC_50_, IC_50_, and *R*^2^ values were estimated from the normalized AChE or BuChE activity with respect to the control assays (absence of the metabolite) and plotted as a function of the logarithm of metabolite concentration using Prism 8 software (GraphPad Software).

## Results and Discussion

In the present study, we aimed to synthesize efficiently four metabolites of Tem described in the literature; two of these (Tem-dox-SO and Tem-dox-SO_2_) were obtained using different Tem:NaClO ratios and two more (Tem-SO and SIDP) via selective oxidation with potassium periodate. It has been assumed that the inhibition of AChE in Tem-treated rats has been mainly attributed to the formation of Tem-active metabolites as a consequence of its biotransformation^14^, since the presence of oxons has not, to our knowledge, been demonstrated, and there is no available toxicological information on other Tem-oxidized metabolites such as Tem-dox-SO, Tem-dox-SO_2,_ and Tem-SO in terms of inhibiting AChE. Therefore, the inhibition potential of human RBC AChE activity by Tem-oxidized metabolites was evaluated. Moreover, by using Tem, the four synthesized compounds and two commercially acquired metabolites (SODP and TDP), we established the chromatographic conditions for the quantitative analysis of all analytes within 15 min.

### Purification of Tem

**Figure 2:**
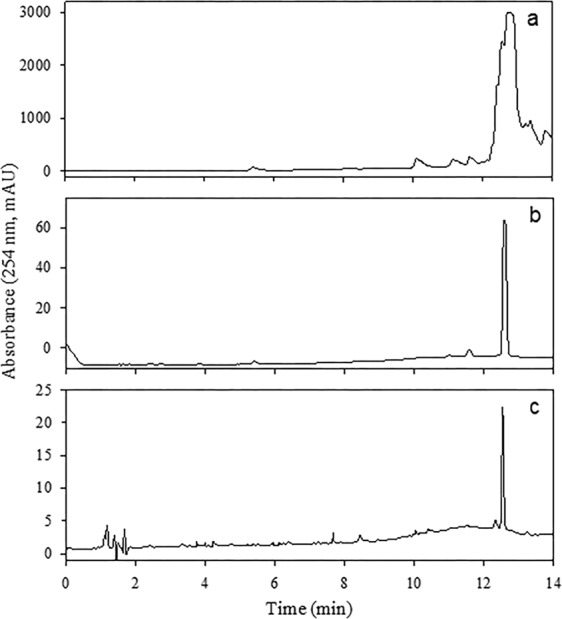
HPLC-DAD chromatogram of T.M. FOS^®^ 500 CE (**a**), Tem purified (**b**), and standard of Tem (**c**) from Sigma-Aldrich Chemical Co. (batch #SZBD207XV).

NMR (Resource 1) and HPLC-DAD (Fig. 2a) analyses of the commercial product T.M. FOS^®^ 500 CE revealed the presence of unknown additives. Tem (*t*_ret_ 12.5 min, yellow oil, 98% purity; Fig. 2b) was purified by evaporation, followed by simple precipitation of the impurities, and a thorough washing with hexane. ^1^H, ^13^C, and ^31^P NMR data (Resource 2) were consistent with previous reports^15^ and were compared to the commercially obtained standard (Fig. 2c).

### Synthesis and identification of temephos oxons

Kamel *et al*.^9^ reported the formation of several Tem-oxidized products when the pesticide was added to chlorinated water. Accordingly, we focused on the large-scale synthesis of two oxidation products, that is, Tem-dox-SO and Tem-dox-SO_2_, using an inexpensive and rapid process by varying the Tem:NaClO ratio. This type of exhaustive oxidation reaction is also common in other organophosphorus pesticides, for which oxon formation has also been observed^9,16,17^.

**Figure 3:**
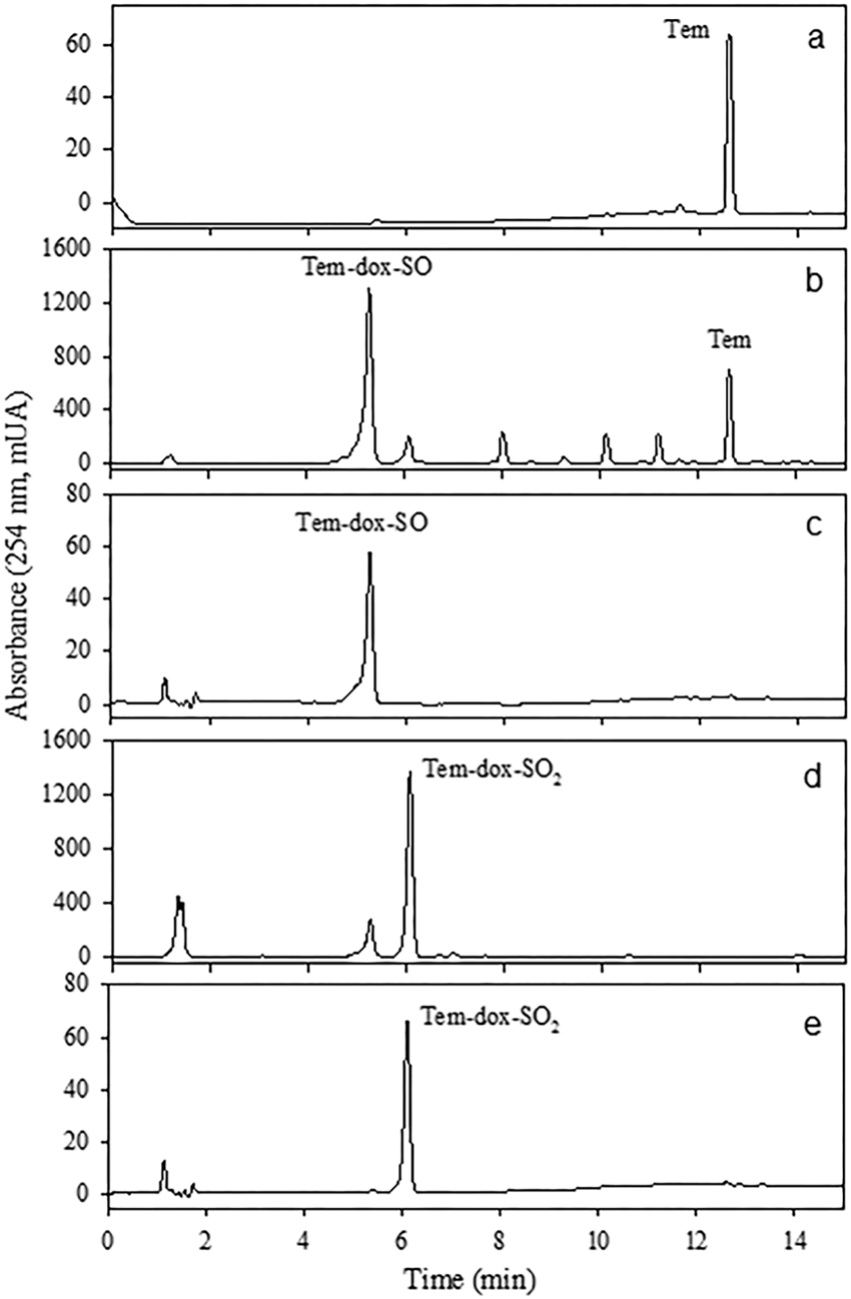
HPLC-DAD chromatograms of Tem and oxidation products by NaClO: Tem purified (**a**); oxidation products of Tem-NaClO (1:10) (**b**); Tem-dox-SO purified (**c**); oxidation products Tem-NaClO (1:100) (**d**); and Tem-dox-SO_2_ purified (**e**).

The HPLC-DAD analysis of the Tem:NaClO 1:10 reaction products is shown in Fig. 3b. The major component (*t*_ret_ 5.2 min, yellow oil, 98% purity; Fig. 3c) was purified by flash chromatography and characterized by NMR (^1^H, ^13^C, and ^31^P), HRMS, and IR. Its molecular formula was deduced as C_16_H_20_O_9_SP_2_ based on the HRESIMS molecular ion peak, which indicated an index of hydrogen deficiency (IHD) of 8 (Δ = −1.1 ppm; Resource 7a). Detailed analysis of the ^1^H NMR data (Resource 3) revealed the presence of a methoxy group (*δ*_H_ 3.85) and four aromatic protons at *δ*_H_ 7.35 (H-3 and H-5) and 7.92 (H-2 and H-6). The ^13^C NMR spectrum (Resource 3) confirmed the presence of six aromatic carbons at *δ*_C_ 121.6 (C-3, C-5), 129.9 (C-2, C6), 138.0 (C-1), and 154.3 (C-4), and one methoxy group at *δ*_C_ 55.2. The ^31^P NMR spectrum (Resource 3) showed a phosphate group (*δ*_P_ −4.9), in agreement with that observed for the methyl-paraoxon (*δ*_P_ −4.8)^18^. Additionally, in the IR spectrum (Resource 8), typical P = O signals, corresponding to the trimethyl phosphine oxide, were observed at 1,190–1,176 cm^−1 ^^19,20^ and at 1,043–1,049 cm^−1^ for the S=O group^21^. Based on these results, the compound was identified as Tem-dox-SO.

Additionally, HPLC-DAD analysis of Tem:NaClO 1:100 reaction products is depicted in Fig. 3d. The minor peak at *t*_ret_ 5.2 min corresponds to Tem-dox-SO, while the major peak at *t*_ret_ 6.0 min (Fig. 3e) was purified and identified as Tem-dox-SO_2_. Briefly, it was isolated as a yellow oil and, with the molecular formula C_16_H_20_O_10_SP_2_ (HRESIMS, Δ = −1.1 ppm, IHD = 8; Resource 7b). The NMR data (Resource 4) suggested a structural similarity to Tem-dox-SO. The key differences were the change in the chemical shift of H2 and H6 (*δ*_H_ 7.61), C2 and C6 (*δ*_C_ 126.6), and C1 (*δ*_C_ 141.7) due to the presence of a sulfone (O=S=O) group. The presence of this group was also supported by the 16 Da difference in the HRMS comparative analysis of this compound and Tem-dox-SO, and by the IR band at 1,153 cm^−1^ (Resource 8)^22^. Finally, the ^31^P NMR spectrum revealed the presence of a phosphate group at (*δ*_P_ −4.6).

Tem-dox-SO and Tem-dox-SO_2_ have only been identified, to our knowledge, in water samples by low-resolution MS; no commercial standards are available^8,9^. The compounds synthesized in this work were characterized by NMR and HRESIMS, and represent the most stable products formed in chlorinated water. Their degree of oxidation was dependent on the concentration of NaClO used in the reaction.

### Synthesis and identification of sulfoxide derivatives

**Figure 4:**
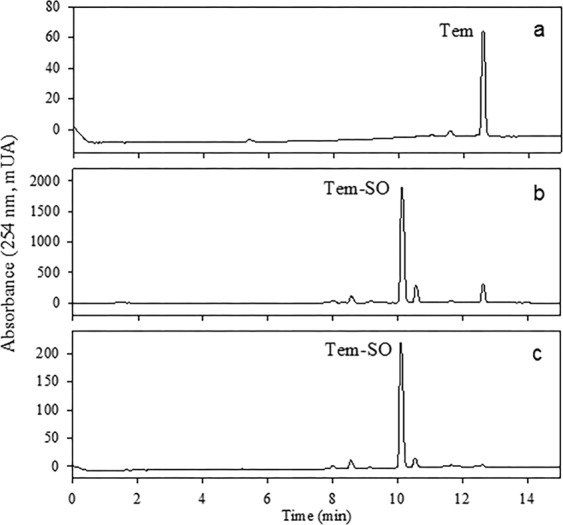
HPLC-DAD chromatograms of Tem and its oxidation products by KIO_4_. Purified Tem (**a**); oxidation products of Tem-KIO_4_ **(**b); and Tem-SO purified (**c**).

**Figure 5:**
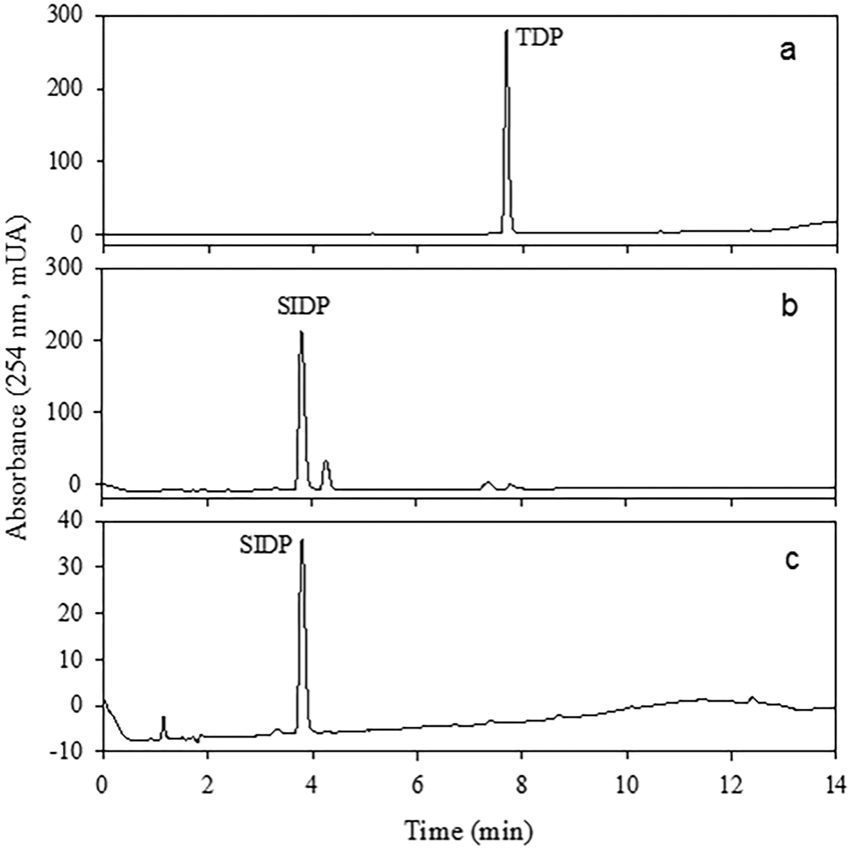
HPLC-DAD chromatograms of thiodiphenol and oxidation products by KIO_4_. Thiodiphenol (**a**); oxidation products of TDP-KIO_4_ (**b**), and SIDP purified (**c**).

Tem-SO, TDP, SIDP, and SODP have been identified in Tem-treated rats^6^, but the toxicological information for most of these in different species is very limited. Since TDP and SODP are commercially available, Tem-SO and SIDP were synthesized utilizing an efficient and low-cost method by the oxidation of Tem and TDP with potassium periodate^21,23^. In Figs. 4b and 5b the chromatograms of extracts obtained from the synthesis of sulfoxides are presented. The major component from Tem (*t*_ret_ 10.2 min, brown oil, ≈98% purity; Fig. 4c) was purified by several washes with hexane/toluene and characterized by NMR (^1^H, ^13^C, and ^31^P), HRMS, and IR. Its molecular formula was deduced as C_16_H_19_O_7_S_3_P_2_ based on the HRESIMS molecular ion peak (IHD = 8, Δ = −0.9 ppm; Resource 7c). The NMR data (Resource 5) suggested a structural similarity to Tem. The key differences were the shift of H2 and H6 (*δ*_H_ 7.71), C2 and C6 (*δ*_C_ 126.4), and C1 (*δ*_C_ 141.1) compared to Tem due to the presence of a sulfoxide (S=O) group, which was evidenced in the IR spectrum at 1,025 cm^−1^ (Resource 9)^15^. Finally, the ^31^P NMR spectrum revealed the presence of a phosphate group at (*δ*_P_ 66.1), similar to that of Tem (*δ*_P_ 66.3)^18^.

In the HPLC-DAD analysis of the extract between TDP with potassium periodate, two additional peaks were observed (Fig. 5b). The major component (*t*_ret_ 3.8 min) was purified by exhaustive washing with hexane/ethyl acetate until reaching a purity of 98% (Fig. 5c) and this was characterized by NMR, HRMS and IR. Briefly, its molecular formula was deduced as C_12_H_10_O_3_S based on HRESIMS data (IHD = 8, Δ = −2.3 ppm; Resource 7d). The ^1^H and ^13^C NMR spectra (Resource 6) exhibited four aromatic protons at *δ*_H_ 7.45 (d, 8.7 Hz, H2 and H6), and 6.91 (d, 8.7 Hz, H3 and H5), and six carbons at *δ*_C_ 127.1 (C2 and C6), 116.0 (C3 and C5), 133.7 (C1), and 160.6 (C4). The difference of 216 and 248 Da compared to Tem-dox-SO and Tem-SO suggested that this compound lacks thiophosphate and phosphate groups. Finally, the presence of a sulfoxide (S=O) group was evidenced by the IR band at 1,010 cm^−1^ (Resource 9).

The synthesis of Tem-SO was described by Leesch and Fukuto^7^ via the several-steps reaction between 4,4′-dihydroxydiphenyl and sulfoxide dimethyl phosphorochlorhydrate. In the present study, we obtained the product of high purity and yield employing a single-step reaction followed by a one-step purification. Moreover, SIDP was also obtained by the simple reaction between TDP and potassium periodate, followed by hexane/ethyl acetate washing. This compound could be an intermediary metabolite produced from the phosphoric diester hydrolysis of Tem-dox-SO and could act as a precursor to form SODP or BPS. BPS is one analog of bisphenol A utilized in the manufacture of polycarbonate feeding bottles. Recent studies on BPS have demonstrated that changes are induced by it in behavior, hepatic metabolism, and serum hormone levels in rats and fishes, and it has also been classified as an endocrine disruptor^24–28^.

### Human cholinesterase inhibition by temephos-oxidized metabolites

**Figure 6:**
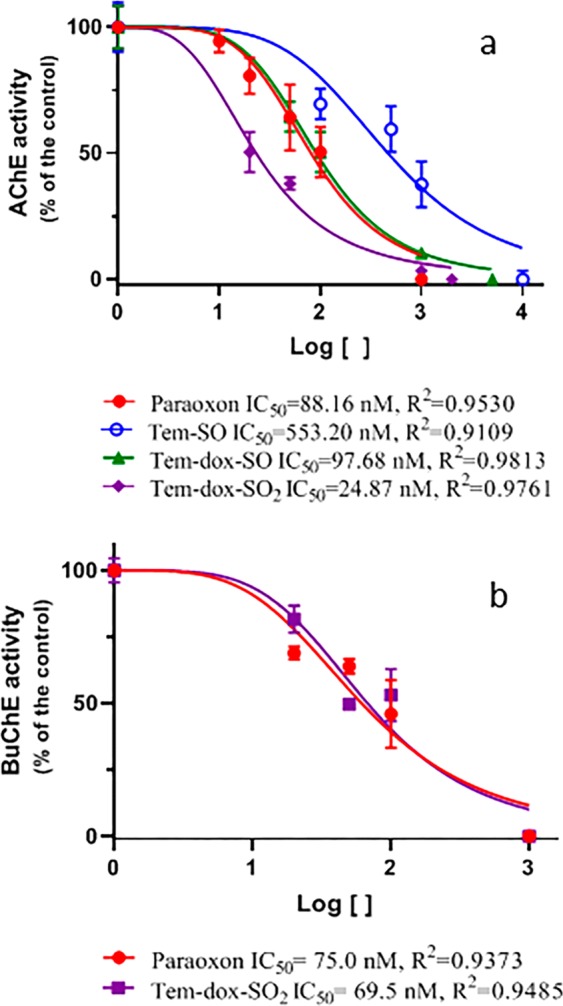
*In vitro* determination of IC_50_ of RCB AChE (**a**) in the presence of Tem-oxidized metabolites and ethyl-paraoxon and BuChE (**b**) in the presence of Tem-dox-SO_2_ and ethyl-paraoxon.

Once oxidized metabolites were chemically identified and purified, it was of fundamental importance to evaluate the potential risk that these compounds could represent to different species. In a context based on the type of insecticide and their possible effects, this was carried out in an *in vitro* assay to evaluate the ability of Tem-oxidized metabolites to inhibit human RBC AChE activity, which could be related with the neurotoxic potential associated with Tem exposure in animal models. Tem did not exhibit any effect on human RBC AChE activity even at higher concentrations than 50 µM. Contrariwise, the oxidized metabolites exhibited a different inhibitory potency on human RBC AChE, which was oxidative status-dependent: Tem-SO (IC_50_ = 553.2 nM) <Tem-dox-SO (IC_50_ = 97.68 nM) <Tem-dox-SO_2_ (IC_50_ = 24.87 nM) (Fig. 6a). Based on the IC_50_ values observed, the Tem-dox-SO_2_ metabolite was the most potent of all Tem metabolites tested. It is noteworthy that the degree of inhibition exhibited by Tem-dox-SO_2_ on RBC AChE was more pronounced than the highly toxic metabolite ethyl-paraoxon used as positive control (IC_50_ = 88.16 nM) (Fig. 6a), which is one of the most neurotoxic organophosphorus pesticides^29^. Considering the relevance of this finding, we decided to examine the inhibitory potential of Tem-dox-SO_2_ on BuChE activity, also using ethyl-paraoxon as positive control. These results show that the inhibitory activity of Tem-dox-SO_2_, the most oxidized product generated by chlorination, is similar to that of ethyl-paraoxon (IC_50_ = 75 nM and 69.50 nM, respectively) (Fig. 6b). The present study represents, to our knowledge, the first report that describes human AChE and BuChE inhibition by Tem-oxidized metabolites, and these results may be relevant to explain the toxic effects described in mammals and other species exposed to Tem^1,14^. Likewise, these results also confirm that Tem needs to be biotransformed in order to inhibit human RBC AChE. Further studies are needed to determine the ability of biotransformation products of Tem to inhibit AChE.

Tem-SO is the main oxidized product in the environment, and is the only one metabolite detected in biological samples exhibited a very low ability to inhibit human RBC AChE. Therefore, the acute toxicity of Tem on the nervous system may be mainly attributed to Tem-oxons as a product of the biotransformation after oxidative desulfuration likely catalyzed by cytochrome P450 (CYP)^14^, as well as to the exposure of Tem-oxidized metabolites generated under oxidative environmental conditions^8^ and enhanced by water chlorination^9^. The *in vitro* results on the inhibition of human RBC AChE by Tem-oxidized metabolites could help clarify the mechanism of action of Tem in mammals, since all of the latter possess the ability to inhibit AChE. Further research is needed on the involvement of CYP in Tem-oxon formation and to evaluate other toxicological properties of Tem-oxidized metabolites. Similarly, these results also suggest the need to re-consider the use of Tem in water destined for human consumption, particularly considering that oxidant environmental conditions and water chlorination favor the rapid formation of potentially toxic Tem metabolites, and to take into account the importance of water monitoring to evaluate exposure to Tem-oxidized residues.

### Liquid chromatography method

With the synthesized compounds in this study (Tem-SO, Tem-dox-SO, Tem-dox-SO_2_, and SIDP) and commercially available metabolites (TDP and BPS), an HPLC-DAD method was developed for the analysis of Tem and its metabolites. In methods previously described for the analysis of Tem that only used Tem and Tem-SO as standards, other metabolites, such as Tem-ox, Tem-dox, and Tem-oxidized metabolites were only determined as degradation products by low-resolution MS^9,15,30^, and Tem hydrolyzed products, such as SIDP, TDP, and BPS, were not considered. These metabolites are important because they are considered the main products in the urinary elimination of Tem^6,7,31^.

**Figure 7:**
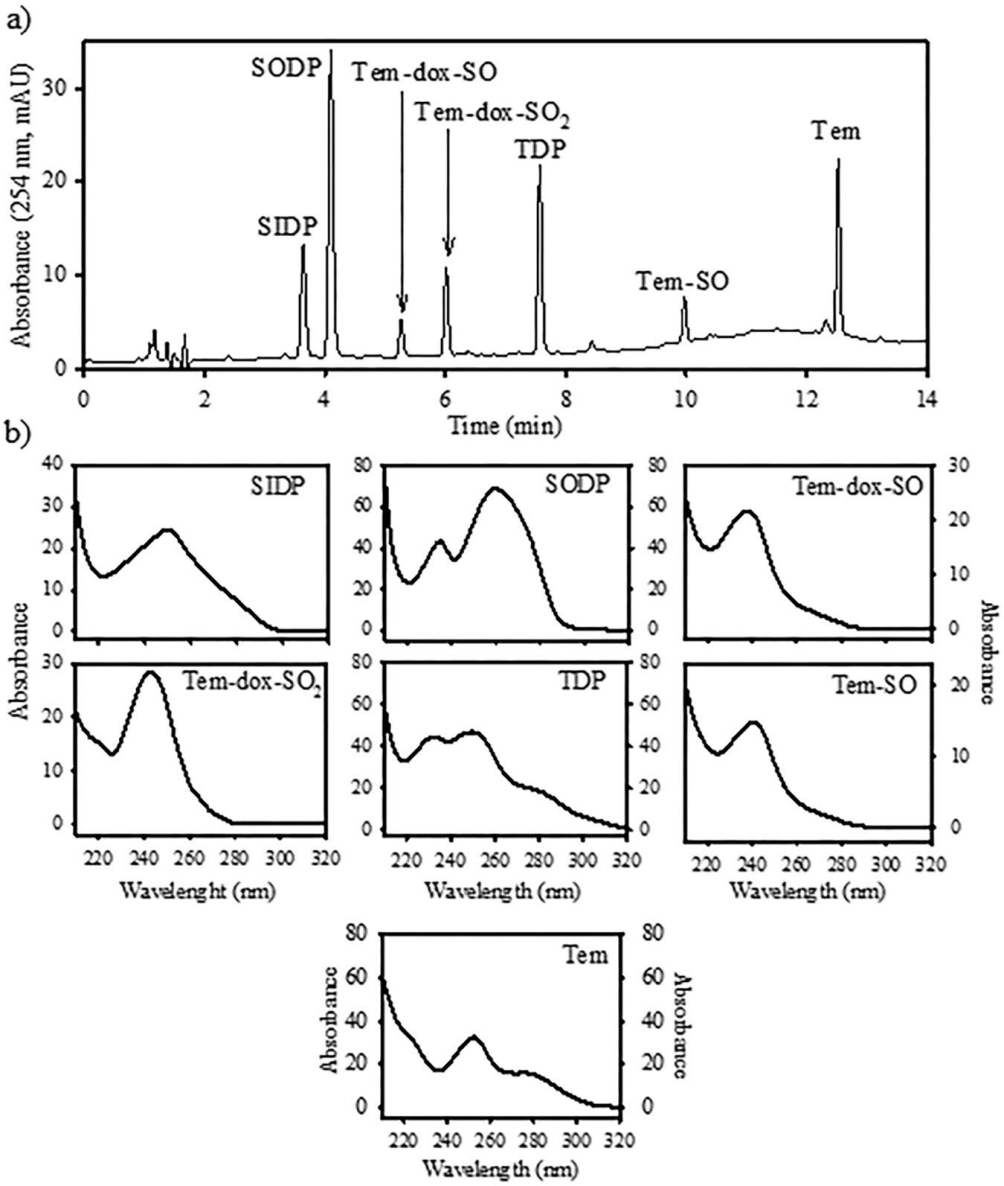
HPLC-DAD chromatogram (**a**) and UV spectra (**b**) of Tem and its metabolites.

Under the chromatographic conditions established, Tem and six metabolites of different polarities were resolved within 15 min with good resolution (Fig. 7). Five-point calibration graphics were performed in methanol from the peak-area measurements for all analytes, revealing linear relationships (*r*^2^ > 0.9876) in a range from 5 to 500 ng (Table 1). Limits of detection (LOD) were calculated from the standard deviation (SD) multiplied by 3.29/slope (calibration curve), and the limits of quantitation (LOQ) from the SD multiplied by 10/slope^32^. LOD and LOQ for all analytes ranged from 0.480 to1.452 and from 1.458 to 4.414 ng, respectively. The SD for each metabolite was calculated by injecting seven replicates of the standard solutions at the lowest concentration of the calibration graphic.

**Table 1:** Limits of detection and quantitation of Temephos and its metabolites.

Compound	*t*_ret_ (min)	Equations	LOD (ng)	LOQ (ng)
SIDP	3.6	y = 1.30x − 3.61	0.849	2.580
SODP	4.1	y = 3.34x − 8.34	0.853	2.593
Tem-dox-SO	5.3	y = 0.97x − 1.92	0.526	1.500
Tem-dox-SO_2_	6.2	y = 1.32x − 3.09	1.155	3.510
TDP	7.7	y = 2.33x − 6.42	1.452	4.414
Tem-SO	10.1	y = 0.92x − 1.20	0.480	1.459
Tem	12.6	y = 1.20x + 3.83	0.917	2.787

The method developed in the present study is the first quantitative method for the monitoring of Tem and six metabolites using authentic analytical standards, which allow us a specific detection of Tem-oxidized metabolites and some of these that could correspond to products of Tem-oxidized metabolite hydrolysis, such as SIDP and SODP or BPS. Compared with other reported methods^9,15^, this analytical method may be useful for the identification and quantification of Tem, of six metabolites, and of other possible metabolites from biological and environmental samples.

In summary, the large-scale synthesis and purification of four Tem oxidation products (Tem-dox-SO, Tem-dox-SO_2_, Tem-SO, and SIDP) were efficiently achieved. The chemical identity of metabolites of Tem was carried out using a combination of conventional spectroscopic techniques. The three oxidized analogs of Tem are potent AChE inhibitors; Tem-dox-SO_2_, the most oxidized and stable product of Tem, is even more potent than ethyl-paraoxon. Furthermore, an HPLC-DAD method for the analysis of Tem and six metabolites was developed; this method may be useful for the determination of Tem and its metabolites in biological and environmental samples. The results of this study suggest high toxicological potential by exposure to Tem. Toxicological information on Tem and its metabolites is limited; therefore, toxicological characterization of Tem and its metabolites is needed for a better understanding of the adverse effects associated with Tem exposure.

## Supplementary information

Verdin-Betancourt et al ESM
