# Supplementary material for: In vitro inhibition of human red blood cell acetylcholinesterase (AChE) by temephos-oxidized products
Source: Sci Rep. 2019 Oct 14;9:14758. doi: 10.1038/s41598-019-51261-2 (PMC6791832; doi:10.1038/s41598-019-51261-2)
Supplement: Supplementary file 1 — Verdin-Betancourt et al ESM [file 41598_2019_51261_MOESM1_ESM.pdf]

In vitro inhibition of human red blood cell acetylcholinesterase (AChE) by temephos-oxidized products

Francisco Alberto Verdín-Betancourt[1], Mario Figueroa[2], María de Lourdes López-González[1], Elizabeth Gómez[3], Yael Yvette Bernal-Hernández[4], Aurora Elizabeth Rojas-García[4], and Adolfo Sierra-Santoyo[1,*]

[1]Departamento de Toxicología, Centro de Investigación y de Estudios Avanzados del Instituto Politécnico Nacional, Col. San Pedro Zacatenco, Ciudad de México C.P. 07360, México.

[2]Facultad de Química and [3]Instituto de Química, Universidad Nacional Autónoma de México, Ciudad de México 04510, México.

[4]Laboratorio de Contaminación y Toxicología Ambiental, Universidad Autónoma de Nayarit, Tepic, Nayarit 63155, México.

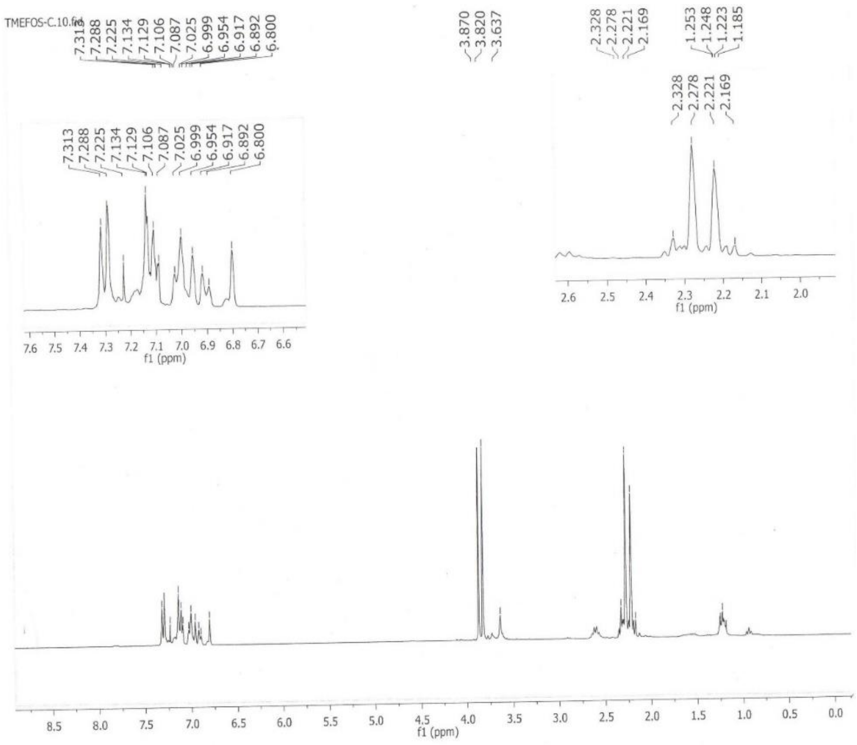

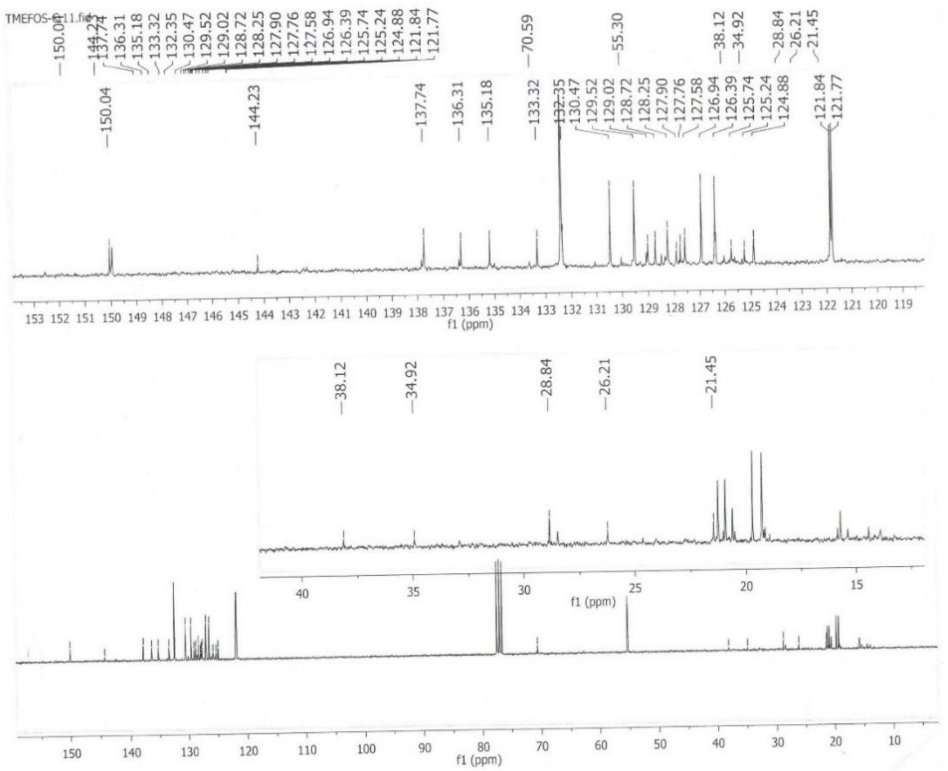

Online Resource 1. ^{1}H (300 MHz; top) and ^{13}C (75 MHz; bottom) NMR spectra of T.M.FOS® 500 CE in $CDCl_3$.

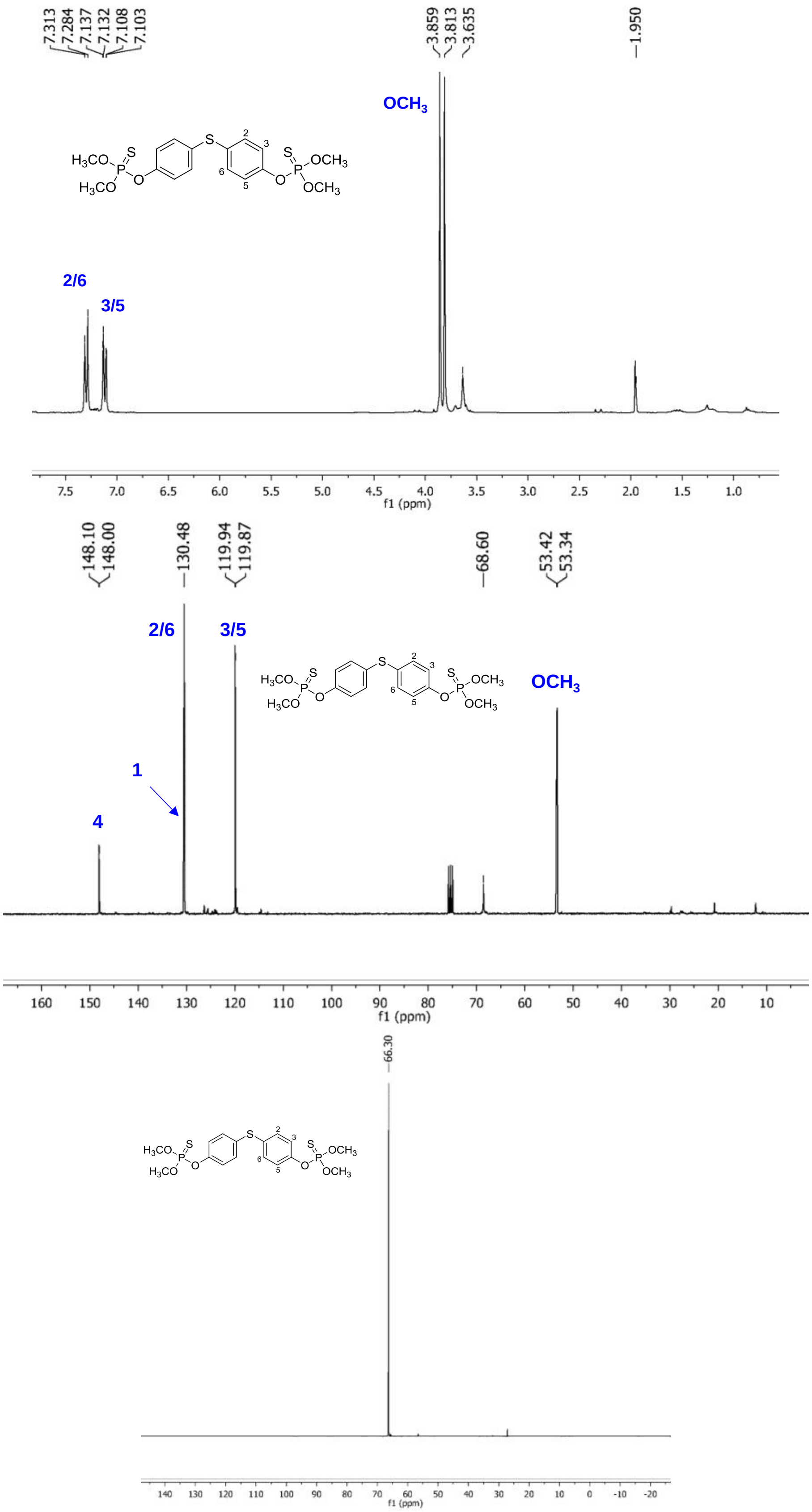

Online Resource 2. ^{1}H (300 MHz; top), ^{13}C (75 MHz; middle) and ^{31}P (121 MHz; bottom) NMR spectra of purified Tem in $CDCl_3$.

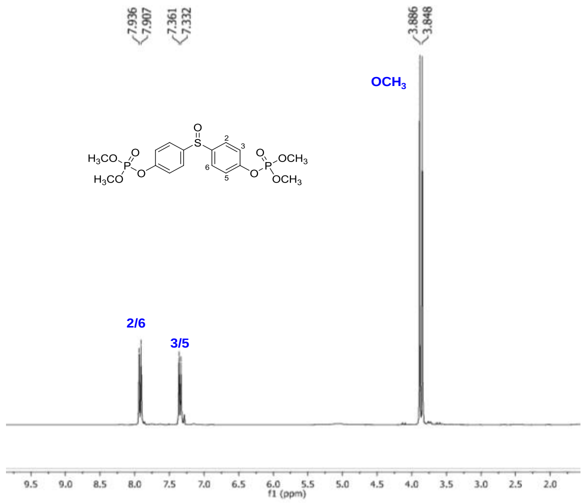

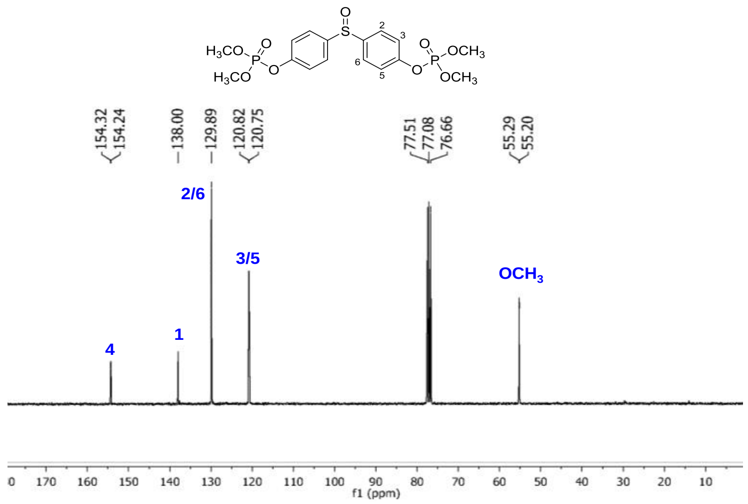

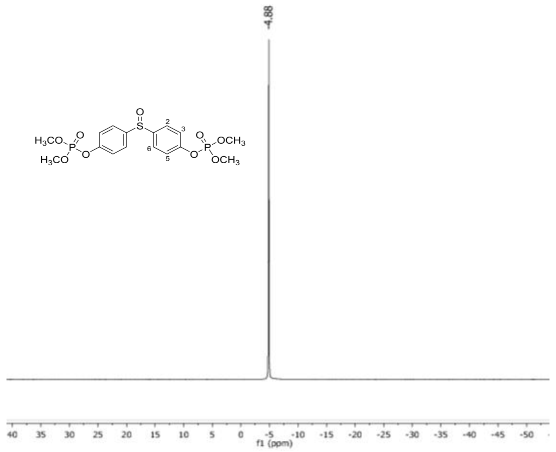

Online Resource 3. ^{1}H (300 MHz; top), ^{13}C (75 MHz; middle) and ^{31}P (121 MHz; bottom) NMR spectra of Tem-diox-SO in $CDCl_3$.

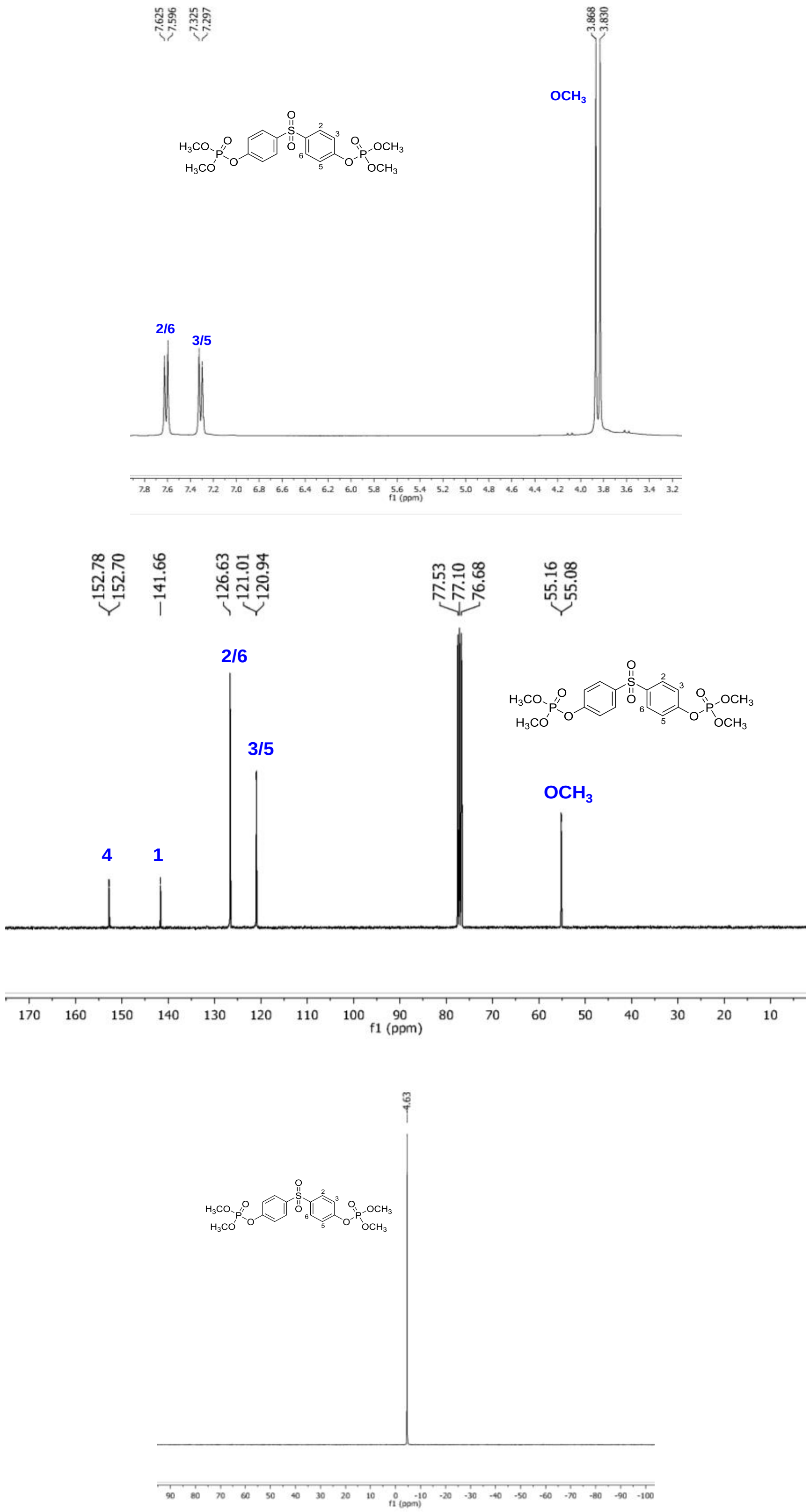

Online Resource 4. ^{1}H (300 MHz; top), ^{13}C (75 MHz; middle) and ^{31}P (121 MHz; bottom) NMR spectra of Tem-diox-SO_2 in $CDCl_3$.

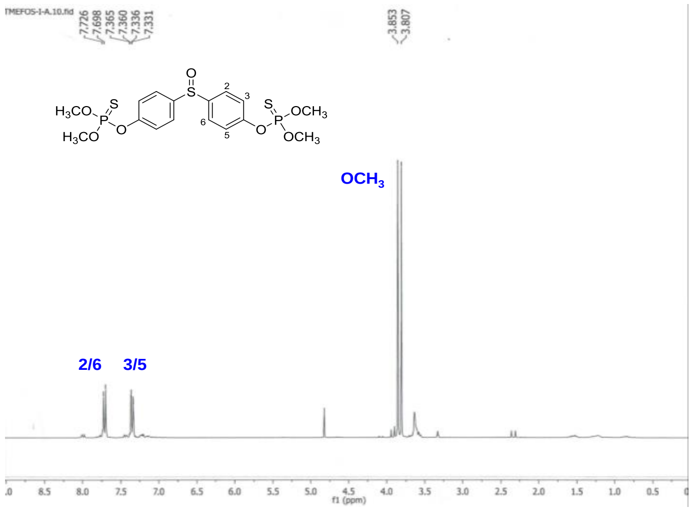

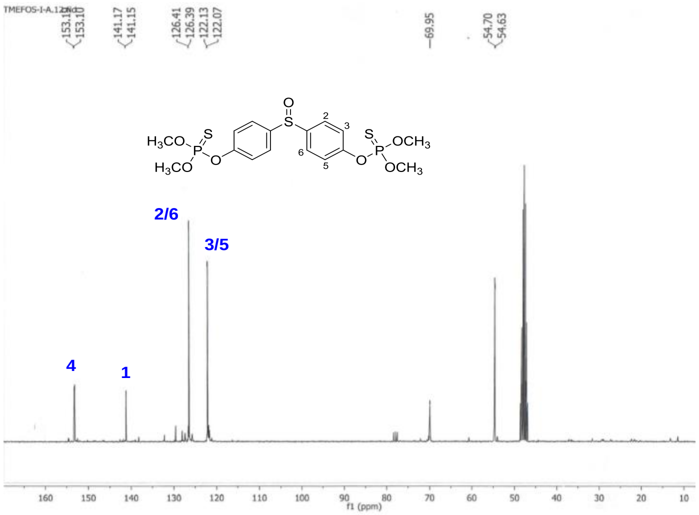

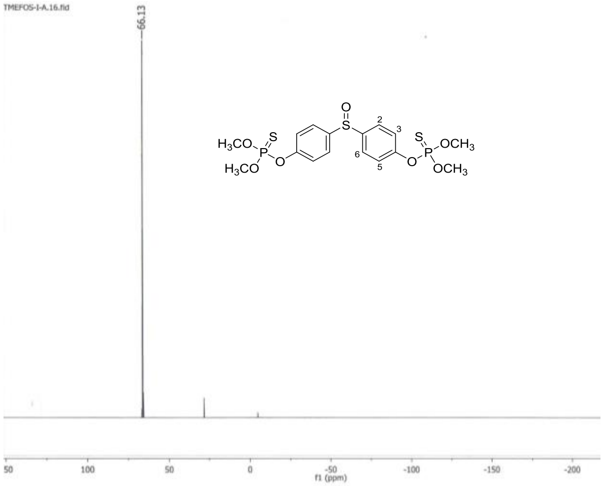

Online Resource 5. ^{1}H (300 MHz; top), ^{13}C (75 MHz; middle) and ^{31}P (121 MHz; bottom) NMR spectra of Tem-SO in MeOH-d_4.

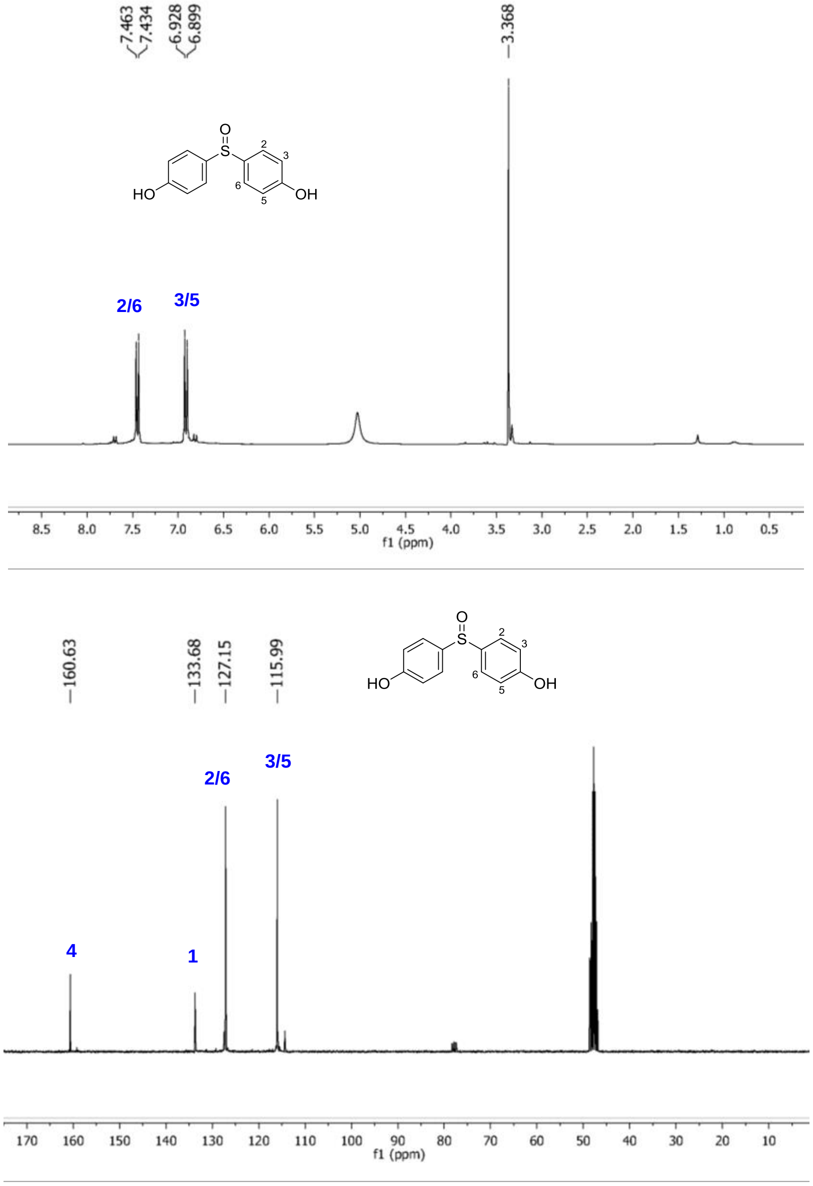

Online Resource 6. ^{1}H (300 MHz; top) and ^{13}C (75 MHz; bottom) NMR spectra of SIDP in MeOH-d_4.

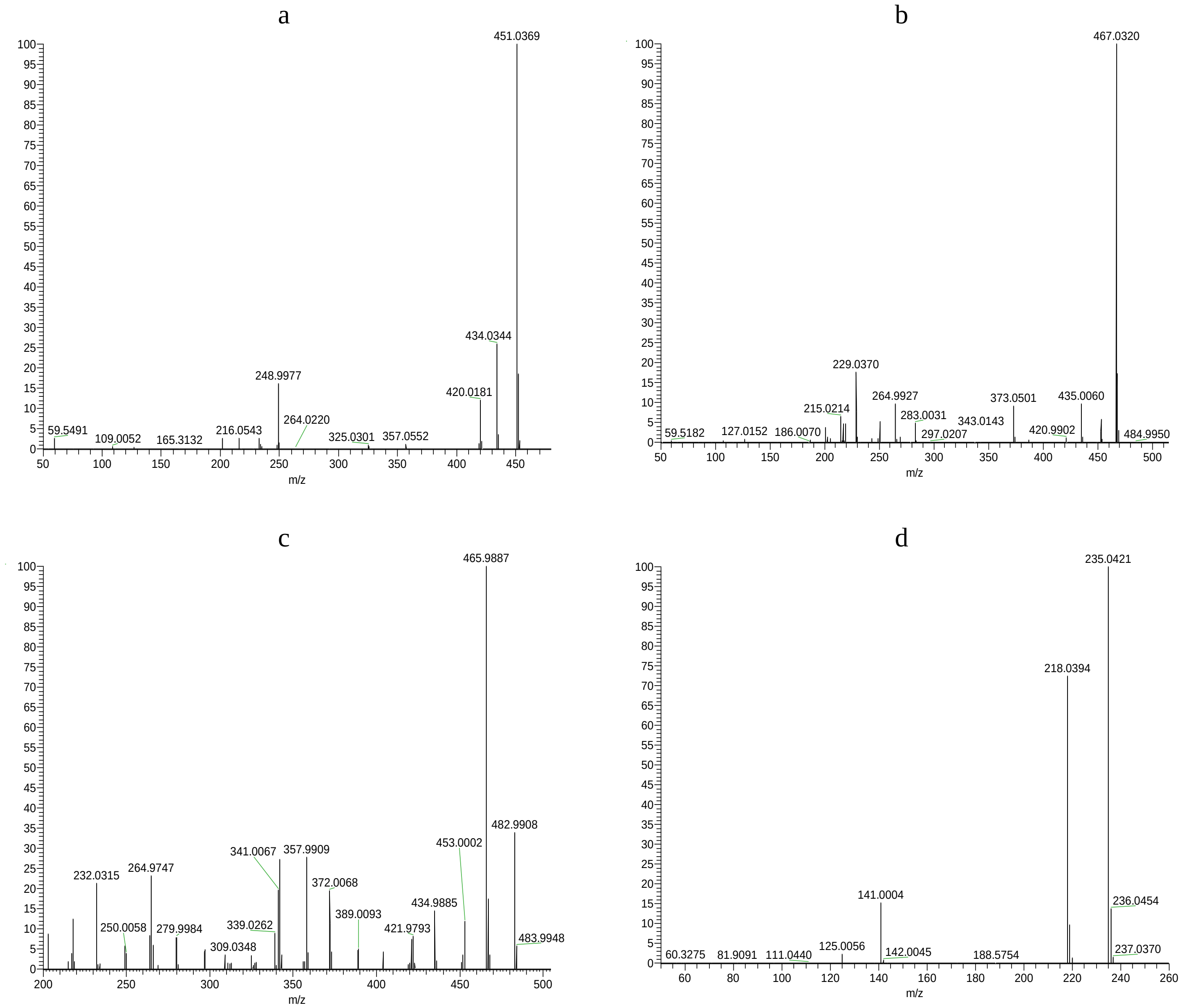

Online Resource 7. HRESIMS (positive mode) spectra of a**)** Tem-dox-SO, b) Tem-dox-SO_2, c) Tem-SO, and d) SIDP.

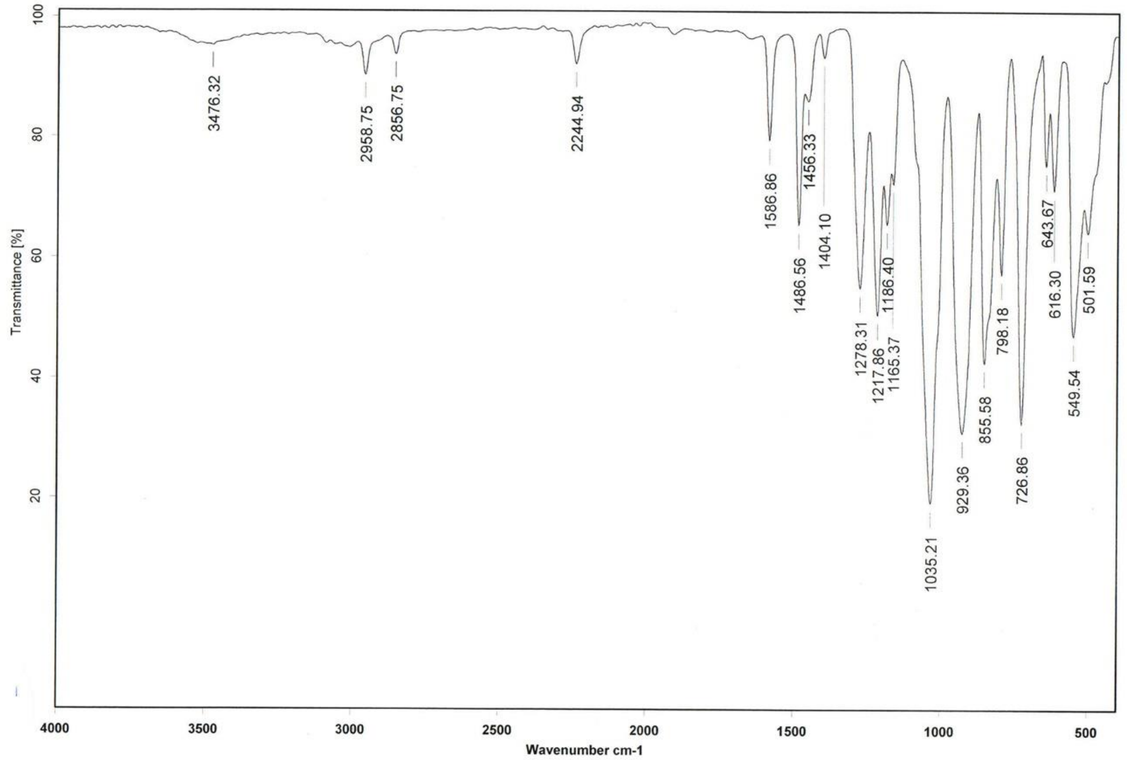

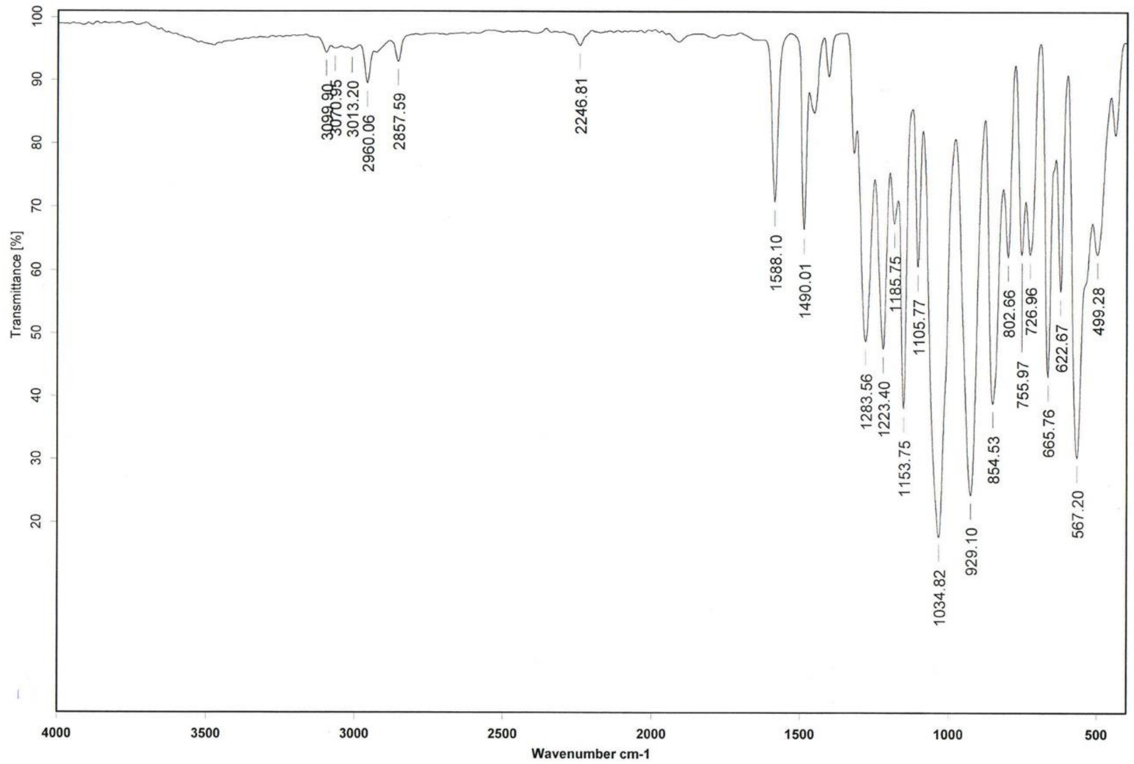

Online Resource 8. FTIR spectrum of Tem-dox-SO (top) and Tem-dox-SO_2 (bottom).

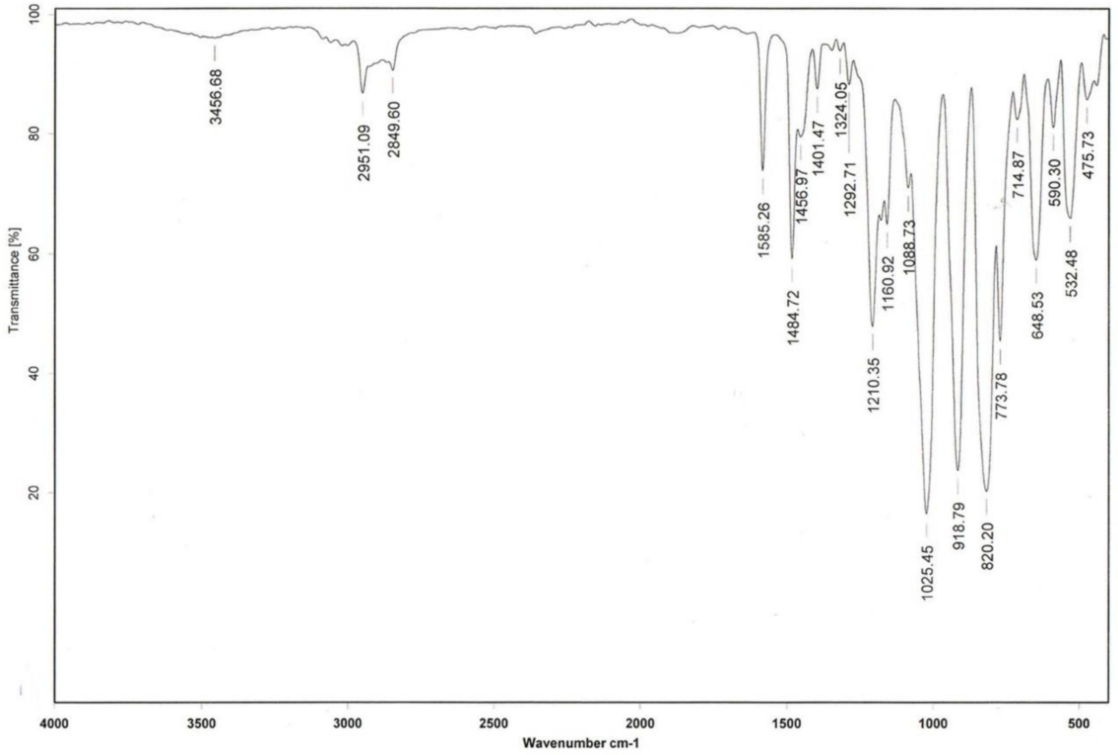

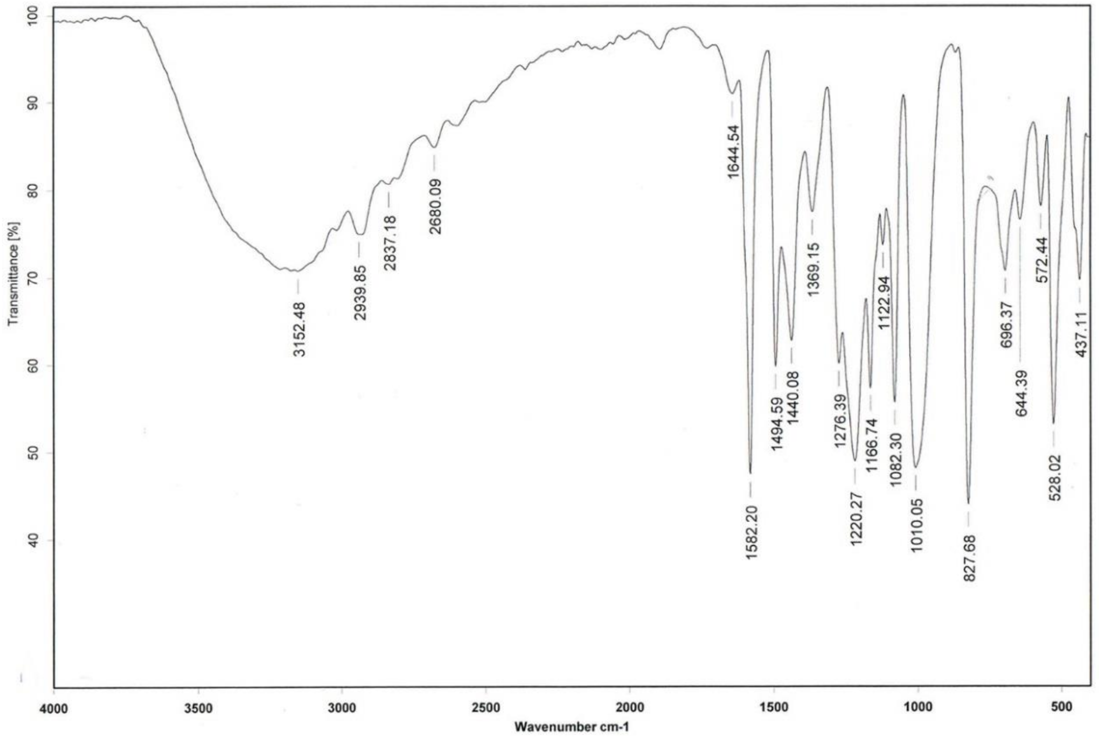

Online Resource 9. FTIR spectrum of Tem-SO (top) and SIDP (bottom).
